# On variability and detecting unreliable measurements in animal cystometry

**DOI:** 10.3389/fruro.2024.1348002

**Published:** 2024-02-13

**Authors:** Zachary C. Danziger, Daniel Jaskowak

**Affiliations:** ^1^ Emory University, Department of Rehabilitation Medicine – Division of Physical Therapy, Atlanta, GA, United States; ^2^ Emory University, W. H. Coulter Department of Biomedical Engineering, Atlanta, GA, United States; ^3^ Florida International University, Department of Biomedical Engineering, Miami, FL, United States

**Keywords:** lower urinary tract (LUT), animal cystometry, cystometry simulation, urodynamic assessment, urodynamic simulation, measurement reliability

## Abstract

**Introduction:**

Animal cystometry, a process of infusing fluid into the urinary bladder to evoke reflex contractions, is a common way to study the effects of pathology, injury, or experimental therapy on lower urinary tract (LUT) dynamics. By monitoring fluid movement during the cystometric micturition cycle, one can compute important quantities that indicate the health and function of the LUT, such as bladder capacity and voiding efficiency. Unfortunately, volume measurements in these difficult studies are often unpreventably corrupted by noise, leading to uncertainty when estimating key cystometric parameters.

**Methods:**

This work proposes a criterion, based on measurable quantities, that flags micturition cycles in cystometry studies that are likely to contain large measurement errors, potentially allowing experimenters to remove them from analysis to obtain a more accurate summary of LUT dynamics.

**Results:**

We describe the criterion, validate it against experimental data, and use computer simulations to demonstrate its utility.

## Introduction

The field-standard for studying animal models of lower urinary tract (LUT) systems physiology continues to be cystometry (1–8). The most common approach is to insert a catheter through the dome of the bladder and infuse physiological saline to induce a bladder distention-evoked reflex contraction (a single micturition cycle). Often cystometry studies are used to compare treatments, such as a pharmaceuticals or nerve stimulation, by computing cystometric parameters that summarize LUT function, such as bladder capacity (BC) or voiding efficiency (VE). To compute the critical cystometric parameters one measures the voided volume ( $V_{V}$ ) and residual volume ( $V_{R}$ ) after each micturition cycle to obtain $BC=V_{V}+V_{R}$ and $VE=V_{V}/\left(V_{V}+V_{R}\right)$ . $V_{V}$ and $V_{R}$ have associated measurement errors that propagate into BC and VE computations, which we strive to minimize to obtain reliable experiment results.

Measuring voided and residual volumes can be difficult (9), and the technical challenges lead to measurement noise and increases in computed cystometric parameter uncertainty. To measure voided volume, it must be collected as it exits the urethra. Often this is done by a collection plate underneath the animal that either rests on a force transducer to record force (and thus weight based on specific gravity) or simply collects the voided volume for it to be subsequently transferred to a measuring device like a graduated syringe. In both cases fluid adhesion to the animal’s fur or urethra, splashing, vibrations, or air bubbles can impact the accuracy of these measurements. The residual volume is harder to measure (10). Often the residual is measured by waiting until the end of a voiding event then withdrawing the remaining fluid from the bladder (through the suprapubic catheter used for bladder infusion) into a graduated syringe (9). The resistance pressure to the manual withdrawal of the residual is a clue for when the bladder is fully emptied (which causes a spike in resistance when the bladder becomes fully collapsed), but if the animal’s abdomen is closed one cannot be certain all the fluid was removed. Due to the detrusor’s pliability, it is possible for it to close around the catheter opening and block the withdrawal before all the residual is actually removed. Moreover, applying too much withdrawal pressure can cause air to seep into the catheter line through a junction with a stopcock. This air displaces fluid in the continuous column between the catheter and bladder and gets withdrawn into the measuring syringe, potentially manifesting as measurement error on future micturition cycles.

Here we propose a conservation of volume principle to flag measurements that are likely to contain higher than usual measurement error, which we hope will lead to an improvement in cystometric technique and more reliable experiment results. This criterion can be used to guide which micturition cycles should be removed from the data set or downweighted when computing group means of cystometric parameters. We show through numerical simulations that cycles that fail to meet the criterion have considerably more cystometric parameter error than those that do. We use a sensitivity analysis to explore which aspects of a cystometry study most affect this accrual of cystometric parameter error, including the effect of bladder infusion rate, and compare the simulations to a cystometry experiment. Ultimately, we hope to improve the fidelity of cystometry studies and curtail exogenous sources of noise in what are experiments with an already high level of intrinsic biological variability (3, 11).

## Methods

We use a simple numerical simulation to model the parameters of individual micturition cycles in a rat cystometry experiment. We assume that for each micturition cycle.

i. Volume is conserved.
ii. The bladder begins the cycle empty.
iii. Volume enters the bladder only through the suprapubic catheter by experimenter-set infusion and naturally via the ureters.
iv. Volume exits the bladder only through the urethra during voiding.

For a given micturition cycle if we stipulate a bladder capacity, BC , a voiding efficiency, VE , an experimentally controlled bladder infusion rate, $Q_{I}$ , and an average ureter inflow rate, $Q_{U}$ , then by conservation of volume (equivalent to conservation of mass in the physiological conditions of the LUT) we can compute what the voided volume $V_{V}$ and residual volume $V_{R}$ must be,


(1) $$V_{V}=VE\cdot t\left(Q_{I}+Q_{U}\right)$$

(2) $$V_{R}=t\left(Q_{I}+Q_{U}\right)-V_{V}$$

This states that the voided volume is defined as the total volume at the time of voiding ( $BC=t\left(Q_{I}+Q_{U}\right)$ ) multiplied by the fraction that was voided, and the residual volume must then be everything that was that was not voided, where t is the duration of the micturition cycle ( $t=BC/\left(Q_{I}+Q_{U}\right)$ ). Decomposing BC into its contributing parts lets us simulate sources of error separately for infused and ureter flows.

To simulate biological variability, each parameter in the model is given an associated mean and spread. To realize a simulated $V_{V}$ and $V_{R}$ for a single micturition cycle, we sample from the VE , BC , $Q_{I}$ , and $Q_{U}$ distributions then compute $V_{V}$ and $V_{R}$ from Equations 1, 2. To simulate measurement error, we add noise to the voided and residual volumes to obtain ${\tilde{V}}_{V}$ and ${\tilde{V}}_{R}$ . Measurement noise is added by drawing a sample from a normal distribution centered on the true parameter value ( $V_{V}$ or $V_{R}$ ) and clipping any negative result at zero (i.e., max{Ν(V,σ),0} , see parameter values and descriptions in **Table 1**). The value sampled from this process is treated as the experimentally measured value ( ${\tilde{V}}_{V}$ or ${\tilde{V}}_{R}$ ).

**Table 1:** Nominal simulation parameters and statistical distributions.

Model Parameter	Mean Value	Units	Distribution	Distribution Parameters	Description
BC	0.25	mL	Truncated Normal	σ=0.05 , range = [0.03, ∞]	Total bladder volume at voiding onset
VE	0.85	–	Truncated Normal	σ=0.15 , range = [0, 1]	Voiding efficiency
$Q_{U}$	0.0033	mL/min	Gamma	k=1.63 , θ=0.002	Ureter inflow rate ( σ=0.0026 )
$Q_{I}$	0.025	mL/min	Truncated Normal	σ=0.001 , range = [0, ∞]	Set pump infusion rate
${\tilde{V}}_{V}$	$V_{V}$	mL	Normal	σ=0.005 , range = [0, ∞]	Voided volume measurement noise
${\tilde{V}}_{R}$	$V_{R}$	mL	Normal	σ=0.05 , range = [0, ∞]	Voided volume measurement noise

For each model parameter, we report its mean value for each simulated micturition cycle, its physical units, the statistical distribution from which it was drawn, the parameters of that distribution, and a brief description of that parameter’s role in the simulation. We use truncated normal distributions (rather than standard normal) because most of the physical quantities in the system cannot meaningfully take negative values. The BC and VE values and their associated distribution values were selected from (12). The $Q_{U}$ , ${\tilde{V}}_{V}$ , ${\tilde{V}}_{R}$ , values and their associated distribution values were selected from (10) (which used seven 267 ± 15g urethane anesthetized female Sprague Dawley rats in open suprapubic fill cystometry). (Note the mean values of voided and residual volume are computed based on BC and VE, not sampled.) The $Q_{I}$ can be changed as needed by experiment and the infusion variability was selected based on internal tests in our lab using the Harvard Apparatus 11 Elite infusion pump. All parameters are estimated from cystometry in Sprague Dawley rats.

From these simulated values we can obtain the experimenter’s error in computing voiding efficiency,


(3) $$E_{VE}=VE-\frac{{\tilde{V}}_{V}}{{\tilde{V}}_{V}+{\tilde{V}}_{R}}$$

Lastly, the experimenter can compute a *post hoc* estimate of the total volume that arrived through the ureters ( ${\tilde{V}}_{U}$ ) using their measurements ( ${\tilde{V}}_{V}$ and ${\tilde{V}}_{R}$ ) and the flow rate command they issued to their infusion pump ( ${\tilde{Q}}_{I}$ ),


(4) $${\tilde{V}}_{U}={\tilde{V}}_{V}+{\tilde{V}}_{R}-t{\tilde{Q}}_{I}$$

which states that the difference between all the total measured volume at the time of the void ( ${\tilde{V}}_{V}+{\tilde{V}}_{R}$ ) and the volume infused by the experimenter ( $t{\tilde{Q}}_{I}$ ) must equal whatever volume the ureters contributed, by the principle of conservation of volume. (Note that ${\tilde{Q}}_{I}\neq Q_{I}$ because the requested infusion rate may not be exactly achieved by the hardware.)

We use ${\tilde{Q}}_{U}$ (i.e., ${\tilde{V}}_{U}/t$ ) as a criterion for flagging micturition cycle measurements that may contain unusually high measurement errors. Since we know that actual volumetric ureter flow ( $Q_{U}$ ) must be non-negative and must be less than some maximum plausible physiological rate (perhaps six expected standard deviations, $\sigma_{Q_{U}}$ , above the expected mean ureter inflow rate, $\mu_{Q_{U}}$ ), if the *post hoc* estimate falls outside this range we flag the micturition cycle as potentially high error. This is expressed as the criterion algorithm in the results.

We updated the nominal simulation parameters to more closely match a particular study (3) to create a direct comparison to experimental cystometry data. The average and standard deviation of VE were updated to the average and standard of all micturition cycles from the data (0.89 and 0.15). The average and standard deviation of BC were updated to the average and standard of all micturition cycles from the data (0.33 and 0.21). The true $Q_{U}$ distribution is not actually measured in the experiment (and cannot be measured), thus the parameters were selected so the resulting simulated *post hoc* flows ( ${\tilde{Q}}_{U}$ ) matched the experiment data, resulting in an updated mean of 0.0098 and standard deviation of 0.0002. The criterion was set to reject micturition cycles if ${\tilde{Q}}_{U}$ was below zero or above 0.0189 for both the simulation and experiment. Cystometry in (3) was run via suprapubic catheter filling in 31 female Sprague Dawley rats under urethane anesthesia (287 ± 28g, 105 ± 15 days old, Charles River Labs).

**Figure 1:**
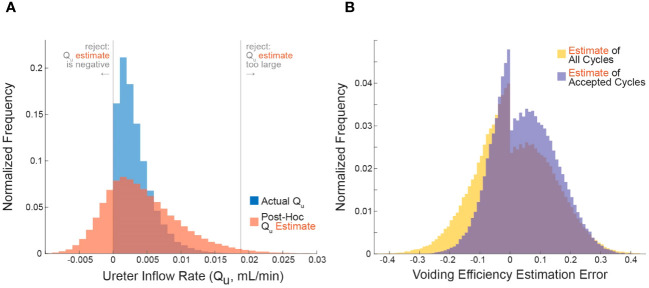
Applying the criterion algorithm in 10^5^ simulations identifies many micturition cycles with very high measurement noise. **(A)** The distributions of actual (blue) and experimenter-estimated (red) ureter inflow rates for all simulated micturition cycles. Vertical lines partition the cycles into which would be rejected or accepted. **(B)** The distribution of errors when estimating VE contains more high-error cycles when considering all cycles (yellow) versus considering only accepted cycles. Negative values are overestimates and positive values are underestimates (Equation 3).

Sensitivity Analysis: We evaluated how robust the criterion was to the chosen parameter values by using a “change one factor at a time” (OFAT) analysis on the estimated voiding efficiency for both criterion-rejected and criterion-accepted cycles. This procedure systematically evaluates the effect of each simulation parameter in isolation on the criterion’s efficacy. To test a set of parameters we take the following steps:

1) Change one of the parameters from its nominal value listed in **Table 1**, leaving all others at their nominal values.
2) Simulate the distribution of voiding efficiency estimation errors for criterion-accepted (**Figure 1B**, purple) and criterion-rejected cycles using the simulation parameters from step-1. Each simulated distribution contained 10^5^ simulated micturition cycles.
3) Represent each distribution as a single number by taking its mean absolute value, which describes the overall error in that distribution.

**Figure 2:**
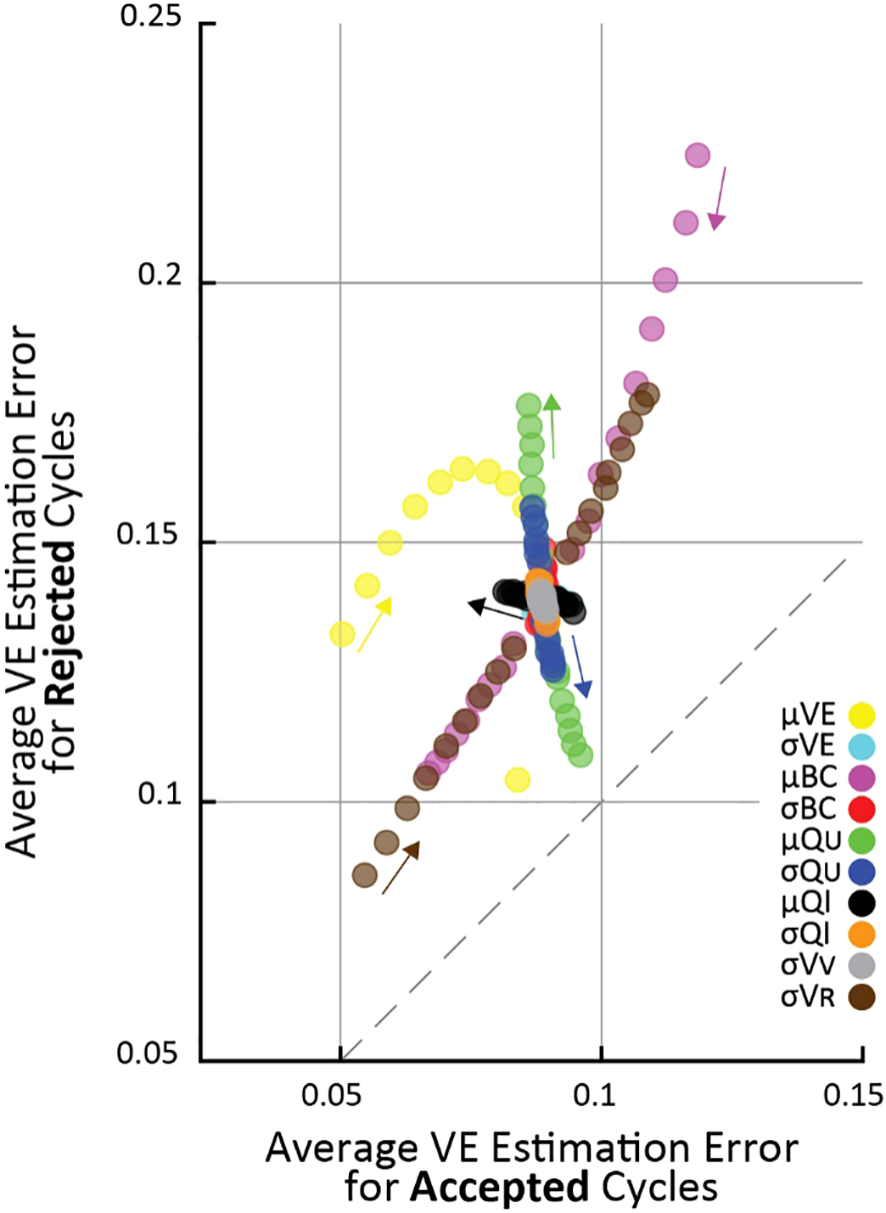
The effect of changing simulation parameters on VE estimation error of cycles passing (horizontal) versus being rejected (vertical) by the criterion in an OFAT analysis. Each point is the average VE error of 10^5^ simulations. Each simulation parameter was changed linearly in 5% increments from 50% above nominal to 50% below nominal, and the parameter that was changed for each set of simulations is indicated by color (arrows point in the direction of increasing parameter value). Fewer μ VE simulations are shown because it had a 0.85 nominal value, and it cannot increase above 1 by definition.

The steps are repeated for every parameter change, resulting in a pair of values for each simulation. **Figure 2** plots these simulations using the two values as the vertical (rejected cycles) and horizontal (accepted cycles) positions for each point. Thus, points above the 45° line show higher estimation error for rejected than accepted cycles.

To systematically evaluate parameter changes, a separate simulation was run with each parameter at -50, -45, -40, -35, -30, -25, -20, -15, -10, 10, 15, 20, 25, 30, 35, 40, 45, 50 percent of their nominal values. For each simulation in **Figure 2**, the changed parameter is indicated by color. Since all parameter changes resulted in smooth changes in VE estimation error, the small arrows show “the direction of increasing parameter value”, meaning the direction VE estimation errors change as the given parameter value is increased.

## Results

We propose a method to flag potentially unreliable measurements in cystometry experiments. The high-level approach is as follows. We assume for a given micturition cycle of duration t that all incoming bladder volume from the ureters and experiment infusion must equal what is ultimately voided out plus the residual volume left behind (Equation 5).


(5) $$t{\tilde{Q}}_{U}+t{\tilde{Q}}_{I}={\tilde{V}}_{V}+{\tilde{V}}_{R}$$

Because we can control the experiment infusion rate, directly observe the duration of the micturition cycle, and measure the voided and residual volume at the end of the cycle, it lets us compute algebraically an estimate of the natural volumetric ureter inflow rate ( ${\tilde{Q}}_{U}$ ). If ${\tilde{Q}}_{U}$ is negative (typically impossible) or larger than some threshold deemed unreasonable, we can flag that micturition cycle as containing potentially unreliable measurements in one (or many) of the blue terms. This is summarized in **Algorithm 1** that is applied to each micturition cycle (where $\mu_{Q_{U}}+6\sigma_{Q_{U}}=0.0189$ mL/min is our estimate for young Sprague Dawley rats, which can be adjusted for species and strains as appropriate).

Algorithm 1 **Criterion algorithm.**

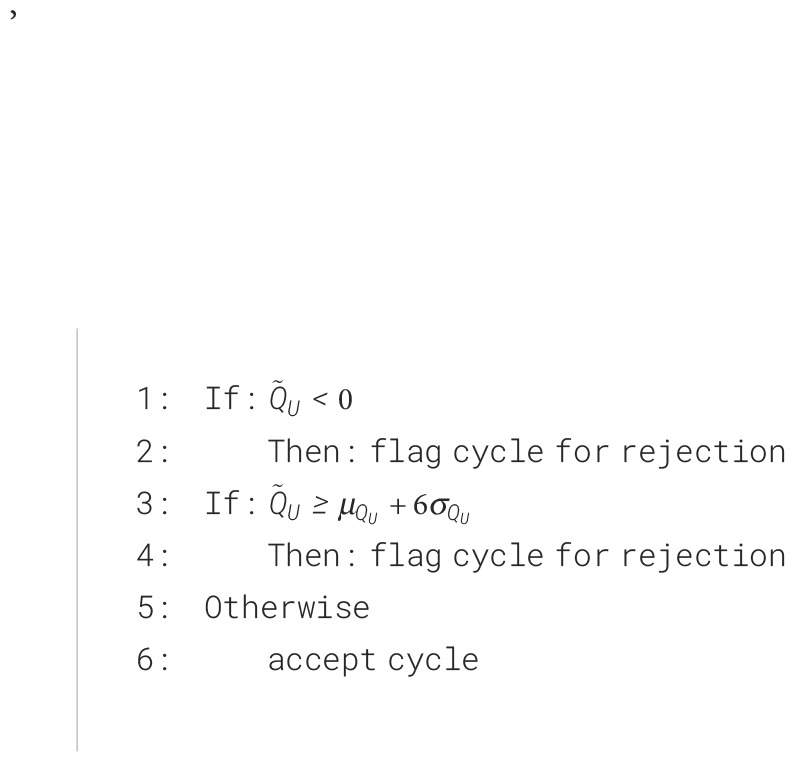

### Simulation of micturition cycles

We simulated 10^5^ micturition cycles by sampling statistical distributions to select a bladder capacity (BC), voiding efficiency (VE), $Q_{I}$ , $Q_{U}$ , and measurement noise for each cycle, then computed the remaining variables by volume balance (see methods for procedure and **Table 1** for parameter values). The blue histogram in **Figure 1A** shows the actual distribution of $Q_{U}$ over all simulated micturition cycles. Note that there are no cycles for which $Q_{U}$ is negative (i.e., fluid flowing back into the kidneys through the ureters). The red histogram in **Figure 1A** shows what an experimenter would estimate $Q_{U}$ to be ( ${\tilde{Q}}_{U}$ ) given their *measurements* of $V_{V}$ and $V_{R}$ and their *expectation* of $Q_{I}$ (not the actual values of these parameters). Note that the ${\tilde{Q}}_{U}$ distribution is much broader than the $Q_{U}$ distribution. The gray vertical lines represent the criterion algorithm for identifying micturition cycles with unreliable data for rejection in the ${\tilde{Q}}_{U}$ distribution. We reject cycles when ${\tilde{Q}}_{U}$ is negative or when ${\tilde{Q}}_{U}$ is implausibly large.

The error in one’s estimate of VE is lower when considering only micturition cycles that are accepted by the criterion algorithm than for all cycles, that is, the criterion can filter out many high-error cycles. **Figure 1B** shows the histogram of errors in computing VE from all micturition cycles (yellow) overlaid against the histogram of VE errors from only those cycles that are accepted by the criterion (purple). The center of mass of the purple distribution is tighter around zero, meaning there is overall lower estimation error in accepted cycles than for all cycles. The average absolute error in estimating VE for cycles passing the criterion is 8.9% (purple), for all cycles it is 10.1% (yellow), and for cycles the criterion rejects it is 13.9% (see **Figure 2**). The discontinuity in the distributions arise because, despite measurement noise, one never experimentally records a negative ${\tilde{V}}_{R}$ ; thus, a VE estimate of 1 becomes disproportionately common, which occurs when ${\tilde{V}}_{R}=0$ .

### Sensitivity analysis

To understand the conditions under which the criterion improves VE estimation accuracy we systematically tested a range of simulated cystometry parameter values; we found that average error for rejected cycles was always higher than for accepted cycles. This is shown in **Figure 2** where each point represents the average of 10^5^ simulated cycles for a given set of simulation parameters (like the data shown in **Figure 1B**), where its vertical position is the average error of criterion-rejected cycles and its horizontal position is the average error of criterion-accepted cycles. The parameter that was changed in each simulation is indicated by color, and the magnitude of change from its nominal (**Table 1**) value is indicated by the small arrows on the plot. For every set of simulations across all parameter changes the average VE error is larger for rejected than accepted cycles (i.e., all points lie above the dashed 45° line).

Most parameters have little effect how well the criterion differentiates high and low error micturition cycles. The notable exception is the average ureter inflow rate ( $\mu_{Q_{U}}$ ); as $\mu_{Q_{U}}$ increases the difference in error between accepted and rejected cycles increases, that is, the criterion is better able to differentially identify high-error estimates. This is visible in the figure by the green points “flowing” perpendicularly away from the 45° line. Other parameter changes affect average VE estimation error, but they do so in a way that impacts both rejected and accepted cycles equally (“flow” parallel to the 45° line), and thus do not have specific implications for the criterion. For example, residual volume ( $\sigma_{V_{R}}$ ) increases error substantially in both accepted and rejected cycles as it grows larger, and average bladder capacity ( $\mu_{BC}$ ) increases error in both accepted and rejected cycles as it becomes smaller.

### Effect of infusion rate on criterion sensitivity

**Figure 3:**
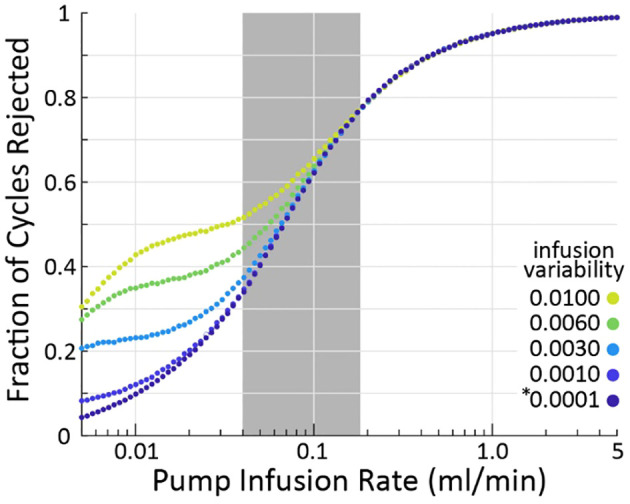
As infusion rate is increased (horizontal), the criterion algorithm will flag more micturition cycles for rejection (vertical), regardless of pump variability (color). The * indicates the nominal infusion variability.

Our simulations show that the larger the infusion rate $\left(Q_{I}\right)$ the more likely the criterion is to reject a micturition cycle, a result born out empirically as well (3). The effect is large, with 10% of cycles rejected at 0.01 ml/min and nearly 100% rejected at 5 ml/min (assuming nominal model parameters, averages over 10^5^ micturition cycles per parameter combination). Greater infusion pump noise (i.e., the difference between the infusion rate the experimenter sets and what the pump actually outputs) also somewhat increases the likelihood of the criterion flagging cycles for rejection. The typical range of infusion rates in rat cystometry (9, 13) is represented by the gray patch in **Figure 3**, indicating that experimenters may expect to find a substantial number of their micturition cycles flagged for rejection (34-77%, although this is likely a slight overestimate as described in the following section). It is important to note that even across this extremely wide range of infusion rates and fraction of rejected cycles, every set of simulations showed larger average VE estimation error for rejected cycles than accepted ones.

### Comparison of simulation to experiments

**Figure 4:**
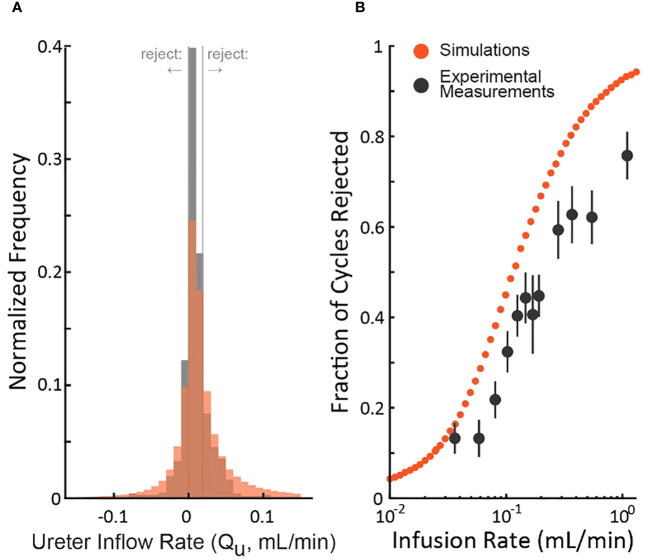
Comparison of the distribution of simulated and experimental *post hoc* ureter flow rate estimate distributions. **(A)** ${\tilde{Q}}_{U}$ distributions from a cystometric study (N=974 micturition cycles, black) and of the simulation using parameters taken from the study (red – color code from **Figure 1**). **(B)** The fraction of rejected micturition cycles as a function of bladder infusion rate from the cystometric study (black, standard errors) and the corresponding simulation (red).

To understand the realism of the simulations, we compared them to an experiment (rat cystometry) that tested a wide range of bladder infusion rates (3) by updating the nominal simulation parameters (**Table 1**) to match those of the study. **Figure 4A** shows the distribution of the experimenter’s *post hoc* estimate of the ureter inflow rate to the bladder as measured by the experiment (black) and simulated (red). The simulation exhibits longer tails and a lower central density than the experiment, but both are normally distributed (Lilliefors statistical test, p>0.5 for both) about similar means. Thus, we expect the simulation to overestimate the number of micturition cycles we would actually reject in the experiment since the longer tails extend farther outside the criterion rejection regions in both directions (vertical lines in **Figure 4A**). The same data are replotted as a function of the bladder infusion rate against the fraction of micturition cycles the criterion rejects in **Figure 4B**. Both simulations and experiment follow a sigmoidal rejection curve with the simulations predicting we would reject on average 16% more cycles at each flow rate than the experiment did.

## Discussion

We described a criterion for identifying which micturition cycles in animal cystometry are likely to have larger than normal measurement errors. The goal is to improve the reliability of these difficult studies by controlling measurement error as a source of added experimental variability. We demonstrated through computer simulations that cycles rejected by the criterion have more estimation error than those accepted (**Figure 1B**), regardless of which simulation parameters are chosen (**Figure 2**). We also estimated the percentage of cycles one should expect to be rejected given the infusion rates used (**Figure 3**), which agreed with experiment results to within 16% (**Figure 4**). Overall, this suggests that the criterion can flag high-error micturition cycles, and perhaps remove them from analysis to improve the reliability of studies.

### Relation between infusion rate and cycle rejection likelihood

Why does increasing the infusion rate increase the probability of flagging a cycle for rejection so substantially? Consider the criteria that if the estimated ureter inflow rate ( ${\tilde{Q}}_{U}$ ) is negative it is flagged for rejection, which we can express from Equation 4 as


(6) $${\tilde{Q}}_{U}=\left({\tilde{V}}_{V}+{\tilde{V}}_{R}-\text{t}{\tilde{Q}}_{I}\right)\frac{1}{t}<\;0$$

where ${\tilde{Q}}_{U}$ is a function of the measured (note the tildes) voided, residual, and infused volume, and t is the total time taken for the complete micturition cycle. If we substitute in Equation 6 the definition of t from Equation 2 and rearrange, we can obtain


(7) $$\frac{{\tilde{V}}_{V}+{\tilde{V}}_{R}}{V_{V}+V_{R}}<\;\frac{{\tilde{Q}}_{I}}{Q_{I}+Q_{U}}$$

which expresses the inequality that must be satisfied to estimate a negative ureter inflow rate. The lefthand side of Equation 7 is independent of infusion rate. On the righthand side, we see that as the infusion rate increases (and thus also the estimated infusion rate) relative to the ureter inflow rate the fraction grows, increasing the likelihood of flagging the cycle for rejection. In other words, the more total bladder volume is contributed by the infusion pump relative to ureters, the more likely one is to find the *post hoc* estimate of the ureter inflow rate to be negative, given the existence of measurement noise.

### Relation of simulations to experiment

Our simulations are a good qualitative match to data from rat cystometry studies (**Figure 4**, both simulated and experimentally measured ${\tilde{Q}}_{U}$ are normally distributed with the same mean), which lends credence to the conclusions we draw from the simulations. Importantly, we must rely on simulations for our argument because we cannot know in experiments which micturition cycles actually have more error (those accepted or rejected by the criterion) since we do not have access to ground truth like we do in the simulations. Thus, we could not determine purely from experiment data if the criterion improves accuracy of accepted cycles. The mismatch between the simulations and the experiment (an overestimate in the number of rejected cycles) is not evidence against the idea or validity of using the criterion since the rejected cycles still have on average higher error than the accepted cycles, even if fewer are rejected than the model predicts *a priori*.

### Use cases

The model was calibrated and validated against rat cystometry studies; however, the same “conservation of volume” logic holds regardless of species and, therefore, the criterion can be used in other animal models. All else held constant, as BC increases (e.g., using a cat instead of a rat) measurement errors on residual and voided volume and pump variability become less relevant compared to this total volume, and the criterion will reject fewer micturition cycles. However, to generate micturition cycles in sufficiently short times for experimental convenience larger infusion rates are necessary. Our simulations show, all else held constant, that increasing infusion rates to maintain a 10 min micturition cycle cancels the benefit of a larger bladder (and this is presuming measurement errors do not increase in larger species, for which we do not have empirical data). When using smaller animal models, like mice, the criterion may reject a very high proportion of cycles due to the small bladder size. Slower infusion rates or some slack in the criterion bounds could mitigate the stringent rejection recommendations. Accordingly, for all cystometry experimental preparations that infuse fluid suprapubically at pressures low enough to avoid generating retrograde ureteral flow the logic of the criterion will hold and can thus be deployed effectively.

The criterion is also valid in models of pathophysiology so long as there is no loss of volume through retrograde flow (or other avenue) and voided and residual volumes are measured. The criterion logic applies when studying detrusor overactivity or the hyperdistended bladder, for example, since Equation 5 still accurately represents the fluid in the different LUT components. The criterion can be used in the study of incontinence as well, so long as all voided volume can be collected throughout the cycle as ${\tilde{V}}_{V}$ . The criterion is not valid for studying dysfunctions caused by fistula because fluid can exit the bladder in a way other than through the urethra, and we lack a term for this in Equation 5 and a means to measure it. Likewise, studying conditions that result in retrograde ureter flow, such as in very high-pressure voiding caused by detrusor-sphincter dyssynergia, invalidate the logic of conservation of volume expressed in Equation 5.

Cases do exist where application of the criterion is unwarranted or not needed. A clear case are preparations that divert ureter flow by cannulation or transection [e.g., see (14)]. The very design of such protocols is to prevent the difficulties that unmeasured renal production introduces in the estimation of cysotmetric parameters; and therefore, no estimation of ureter inflow rate is required and the criterion is not applicable. Though even in this case, one still expects volume measurements to balance with infused volume, i.e. ${\tilde{V}}_{V}+{\tilde{V}}_{R}=\text{t}{\tilde{Q}}_{I}$ , so a version of this criterion would still apply. In isovolumetric cystometry [where the urethra is occluded by, say, a ligature, e.g. see (15)] often no measurements of residual volume are taken, but instead the experimental focus is on the pressure generated by the detrusor. Therefore, we lack the information required by Equation 5 to compute the criterion. The serial cystometrogram [continuous uninterrupted bladder filling across many micturition cycles (1)] presents a similar challenge because the residual volumes are not measured following each void due to the requirement of uninterrupted filling, and thus we again lack the data required by Equation 5. However, algorithms are now available to accurately estimate all residual volumes in serial cystometry, provided the bladder begins empty and the residual of the final void is measured (10, 12); therefore, even for serial cystometry the criterion can be applied provided the data are properly gathered to run the residual estimation algorithm.

### Practical implementation

It is straightforward to apply this criterion to cystometry data in two steps. 1) For each micturition cycle, one can plug in the experimentally measured voided volume, residual volume, time to void, and requested catheter infusion rate into Equation 5 to compute the estimated ureter inflow rate, ${\tilde{Q}}_{U}=\left({\tilde{V}}_{V}+{\tilde{V}}_{R}-t{\tilde{Q}}_{I}\right)/t$ . 2) Use the criterion algorithm to estimate which cycles have unusually large measurement noise, and remove them from analysis.

Practically speaking for cystometry experiments, our results indicate more micturition cycles should be recorded than normal, so that those flagged for rejection can be eliminated without dropping the total sample size below the threshold required for statistically robust analysis. The remaining accepted micturition cycles will yield a more accurate estimate of the cystometric parameters under study. Collecting many replicates is especially important when using larger infusion rates, since we show this regime spreads ${\tilde{Q}}_{U}$ estimates farther into negative values, thus prompting more rejections and making the criterion harder to implement. Lastly, we note that changing the upper cutoff on ${\tilde{Q}}_{U}$ in the criterion to reject micturition cycles has very little effect on how many cycle rejections one expects because most are rejected due to violating the negativity bound.

## Data availability statement

The original contributions presented in the study are included in the article/**Supplementary Material**. Further inquiries can be directed to the corresponding author.

## Author contributions

ZD: Conceptualization, Formal analysis, Funding acquisition, Investigation, Methodology, Project administration, Resources, Software, Supervision, Validation, Visualization, Writing – original draft, Writing – review & editing. DJ: Conceptualization, Data curation, Writing – review & editing.
